# Role of scattering and birefringence in phase retardation revealed by locus of Stokes vector on Poincaré sphere

**DOI:** 10.1117/1.JBO.25.5.057001

**Published:** 2020-05-20

**Authors:** Mariia Borovkova, Alexander Bykov, Alexey Popov, Igor Meglinski

**Affiliations:** a University of Oulu, Optoelectronics and Measurement Techniques Research Unit, Oulu, Finland; b VTT Technical Research Centre of Finland, Oulu, Finland; c National Research Tomsk State University, Interdisciplinary Laboratory of Biophotonics, Tomsk, Russia; d National Research Nuclear University “MEPhI”, Institute of Engineering Physics for Biomedicine (PhysBio), Moscow, Russia; e Aston University, School of Engineering and Applied Science, Birmingham, United Kingdom; f Aston University, School of Life and Health Sciences, Birmingham, United Kingdom

**Keywords:** optical polarimetry, birefringence, scattering, Poincaré sphere, skin, tissue phantoms

## Abstract

**Significance:** Biological tissues are typically characterized by high anisotropic scattering and may also exhibit linear form birefringence. Both scattering and birefringence bias the phase shift between transverse electric field components of polarized light. These phase alterations are associated with particular structural malformations in the tissue. In fact, the majority of polarization-based techniques are unable to distinguish the nature of the phase shift induced by birefringence or scattering of light.

**Aim:** We explore the distinct contributions of scattering and birefringence in the phase retardation of circularly polarized light propagated in turbid tissue-like scattering medium.

**Approach:** The circularly polarized light in frame of Stokes polarimetry approach is used for the screening of biotissue phantoms and chicken skin samples. The change of optical properties in chicken skin is accomplished by optical clearing, which reduces scattering, and mechanical stretch, which induces birefringence. The change of optical properties of skin tissue is confirmed by spectrophotometric measurements and second-harmonic generation imaging.

**Results:** The contributions of scattering and birefringence in the phase retardation of circularly polarized light propagated in biological tissues are distinguished by the locus of the Stokes vector mapped on the Poincaré sphere. The phase retardation of circularly polarized light due to scattering alterations is assessed. The value of birefringence in chicken skin is estimated as $0.3\times 10^{-3}$, which agrees with alternative studies. The change of birefringence of skin tissue due to mechanical stretch in the order of $10^{-6}$ is detected.

**Conclusions:** While the polarimetric parameters on their own do not allow distinguishing the contributions of scattering and birefringence, the resultant Stokes vector trajectory on the Poincaré sphere reveals the role of scattering and birefringence in the total phase retardation. The described approach, applied independently or in combination with Mueller polarimetry, can be beneficial for the advanced characterization of various types of malformations within biological tissues.

## 1 Introduction

The use of polarized light in various biomedical applications is rapidly growing in the recent years.^1^ The advantages of polarization-based diagnostic modalities over the conventional optical techniques, as well as the features and challenges of the characterization of biological tissues using so-called optical polarization fingerprint, are widely described elsewhere.^2^^–^^5^ Due to its unique properties, the polarized light is widely used as a considerable add-on to a number of conventional diagnostic and imaging techniques. This addition provides valuable insight on morphological structure of a biotissue. The examples are polarization-sensitive optical coherence tomography,^6^ polarization-sensitive hyperspectral imaging,^7^ second-harmonic generation (SHG) polarimetry,^8^ polarization-sensitive microscopy,^9^ and others.

The field-based Jones-vector formalism and the intensity-based Stokes–Mueller calculus are the two major mathematical approaches that define the state of polarization of light and describe interaction of polarized light with media.^10^ A number of innovative polarization-based modalities have been developed for various biomedical applications, utilizing physically measurable Stokes–Mueller parameters, e.g., Mueller-matrix polarimetry.^11^^–^^14^ This approach enables obtaining a complete 4×4 Mueller matrix of the sample that contains full polarimetric information of the examined sample. Mueller-matrix polarimetry shows promising results, in particular, in screening of cancerous tissues^15^^–^^18^ and characterization of other turbid tissue-like scattering media.^19^^–^^21^ Moreover, it has been demonstrated that utilizing circularly polarized light in frame of Stokes-vector polarimetry approach, complemented by the use of Poincaré sphere as a quantitative graphical tool, has a high potential for tissue characterization and evaluation of cancer aggressiveness.^22^^–^^27^

In terms of optical properties, besides absorption, biological tissues are characterized by scattering (typically, in the order of tens of ${\mathrm{mm}}^{-1}$ for visible light) and high anisotropy of scattering (g≈0.8).^28^ In addition to scattering of light, due to heterogeneous fibrous structure, biological tissues often exhibit linear form birefringence, which is a measurable quantity; its changes may act as a metric for certain structural abnormalities of biological tissues.^29^ In fact, both scattering and birefringence may elaborate phase shift between electric field components of the field vector of polarized light during its propagation within the biological medium. Examples of such tissues, cartilage^30^ or tendon,^31^ exhibit sufficient form birefringence due to linearly ordered structures,^4^ and the abnormalities of the structure can be detected fairly easily. On the contrary, skin collagen fibers are characterized by arbitrary orientation,^32^^,^^33^ which makes the birefringence contribution to the phase shift between electric field components of polarized light very minor compared to scattering. This poses a challenge to distinguish birefringence in skin and analyze its changes due to possible structural abnormalities of the tissue. Thus, in the frame of Mueller-matrix polarimetry, it is not possible to distinguish the phase shift between transverse electric field components occurring due to birefringence from one taking place due to light scattering. The aim of the current study is to explore how the variations of birefringence and scattering contribute to the overall phase retardation of circularly polarized light propagated in turbid tissue-like scattering medium, such as skin. We apply laser-based Stokes-vector polarimetry with circularly polarized illumination,^22^ which is a robust and more cost-effective approach for the tissue characterization than the Mueller-matrix polarimetry. This laser scanning imaging approach ensures better control of light localization within the tissue sample. The advantages of circularly polarized light include directional awareness,^34^^–^^37^ i.e., the flip in helicity in case of backscattering and helicity preservation for forward scattering. This phenomenon, known as the polarization memory of circularly polarized light,^34^^,^^35^^,^^38^ is of fundamental importance. Linear polarization possesses no such sense of the direction in which light travels.

In order to systematically investigate the alterations of phase shift between transverse components of circularly polarized light due to scattering and birefringence, we utilize both the phantoms of biological tissues fabricated in-house and tissue samples. The chicken skin was chosen as an example of biological tissue due to the presence of both form and intrinsic birefringence inherent to collagen^39^^,^^40^ as well as scattering.

## 2 Methods and Materials

### 2.1 Experimental System

**Fig. 1:**
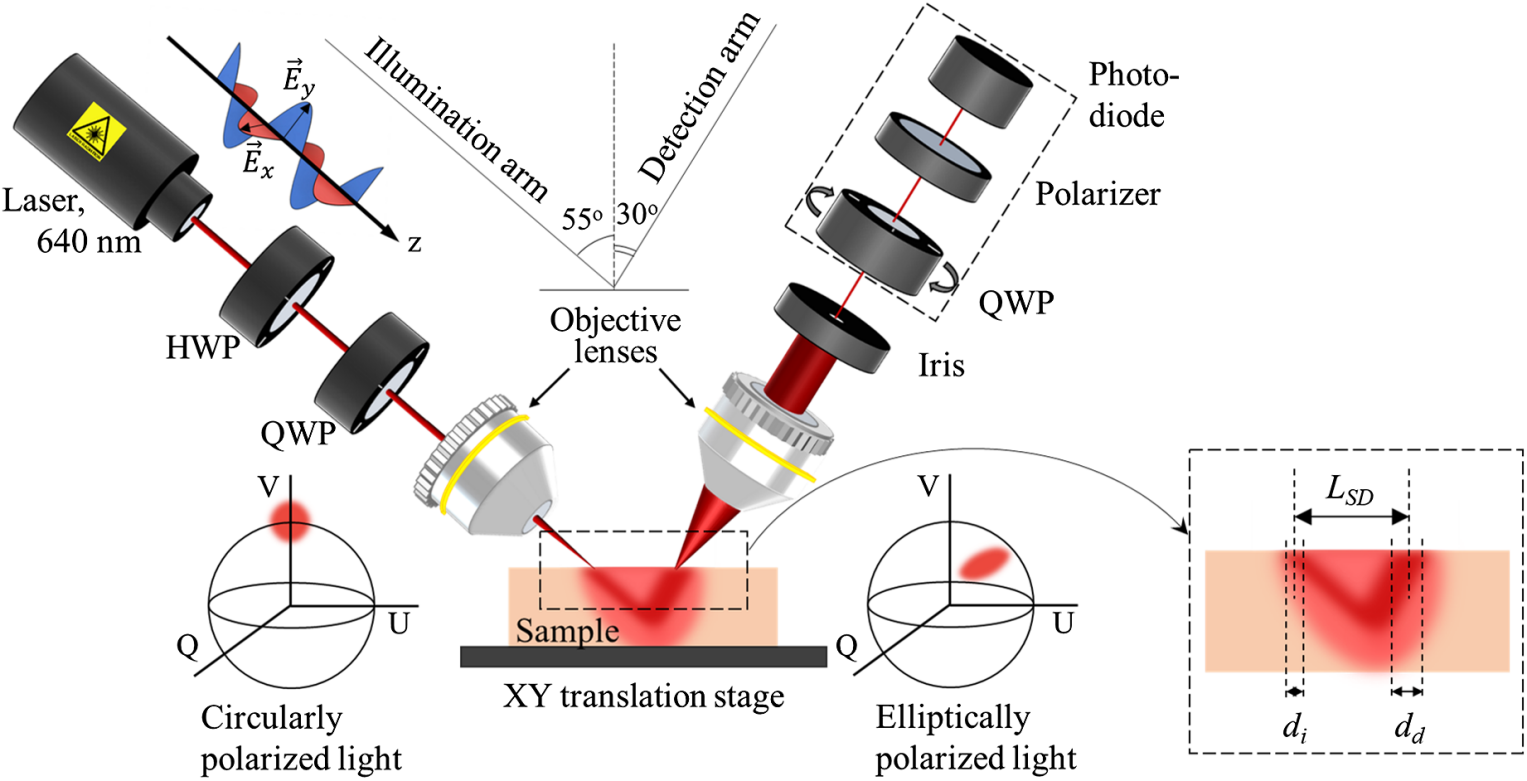
The schematic presentation of the experimental setup. Inset shows incident and detection spots and $L_{\mathrm{SD}}$. Explanations are given in the text.

In the experimental system (Fig. 1) developed in-house, the linearly polarized light produced by a laser source (640 nm, Edmund Optics) was altered by half-wave and quarter-wave plates into the right-hand circular polarization. The right-hand circularly polarized light was focused with an objective lens on the sample at 55 deg angle. The sample was placed on the X–Y translation stage. The backscattered light was collected with an objective lens at 30 deg angle at a variable distance $L_{\mathrm{SD}}$ away from the point of incidence, vignetted by a 100-μm iris and its state of polarization was analyzed by the Stokes-vector polarimeter (Thorlabs), which consisted of a rotating quarter-wave plate, a polarizer, and a power meter. The diameter of the incident focused laser beam $d_{i}$ was ∼15  μm [measured with a laser beam profiler (BeamMaster BM-7, Coherent)]. The field of view of the objective lens in the detection arm $d_{d}$ was 50  μm. The measured Stokes vectors were analyzed using the Poincaré sphere as a graphical tool.^22^^,^^26^^,^^27^^,^^41^ In Fig. 1, two Poincaré spheres show, respectively, the position of the Stokes vector of incident right circularly polarized light (sphere on the left) and its relative changes upon interaction with the medium/tissue sample (sphere on the right). The described experimental system has been used extensively in previous studies.^22^^,^^23^^,^^25^

### 2.2 Experimental Protocols

In order to explore the contributions of scattering and birefringence to the phase retardation of circularly polarized light, a series of experiments with biotissue-mimicking phantoms and biological tissues has been performed.

#### 2.2.1 Model experiments

*Variation of source–detector separation*. In order to confirm the impact of the source–detector separation $L_{\mathrm{SD}}$ on the state of polarization of light scattered from a turbid tissue-like scattering medium, an experiment with variation of the source–detector separation was performed utilizing a tissue phantom. The state of polarization of light scattered from the phantom ($\mu_{s}=6\;{\mathrm{mm}}^{-1}$, g≈0.8, and thickness=8  mm) was measured with different source–detector separations ($-0.05\;\mathrm{mm}\leq L_{\mathrm{SD}}\leq 0.7\;\mathrm{mm}$). The value of $L_{\mathrm{SD}}$ was measured from the estimated zero point, which was the place of coincidence of focal points of the illumination and detection arms, which corresponded to the highest intensity on the detector. The point of coincidence was set as $L_{\mathrm{SD}}=0$; further convergence of the source and detector was considered negative $L_{\mathrm{SD}}$, while their separation was considered positive $L_{\mathrm{SD}}$.

Tissue-mimicking phantoms with the confirmed optical properties at certain wavelengths were fabricated in-house from polyvinyl chloride plastisol (M-F Manufacturing Co.), a white opaque fatty solution of monomers that polymerizes and becomes transparent at high temperatures. ZnO particles (Sigma-Aldrich, Germany) were used to imitate scattering properties assessed based on concentration and size distribution retrieved from the scanning/transmission electron microscopy. The preparation procedure was described elsewhere.^42^^,^^43^ The fabricated phantoms were stored on glass slides at room temperature protected from direct light. The scattering properties of the tissue phantoms were confirmed with the standard measurements of collimated transmittance, total transmittance, and total reflectance^43^^,^^44^ using the spectrophotometric system equipped with integrating spheres OL-750 (Optronic Laboratories) in 600- to 700-nm spectral range. The thickness of samples was measured with the optical coherence tomograph (Hyperion, Thorlabs), whereas the refractive index was estimated with the Abbe refractometer (DR-M2 1550, Atago, Japan).

*Alteration of phase of circularly polarized light due to scattering and birefringence*. In the model experiments, the change of scattering was achieved by utilizing tissue phantoms with different scattering coefficients ($\mu_{s}=4$ and $8\;{\mathrm{mm}}^{-1}$, g≈0.8, thickness=1  mm), whereas the phase alteration occurring due to birefringence was mimicked through adding a variable phase shift into incident illumination utilizing the half-wave plate (see Fig. 1). Experiments were performed with $L_{\mathrm{SD}}=1.5\;\mathrm{mm}$. In order to demonstrate the phase alterations due to birefringence in the absence of scattering, a simple experiment with a mirror used as a sample was performed ($L_{\mathrm{SD}}=0\;\mathrm{mm}$ and $\mu_{s}=0\;{\mathrm{mm}}^{-1}$; the angles of incidence and detection were changed in order to detect reflection from the mirror).

#### 2.2.2 Distinguishing scattering and birefringence in phase alterations using chicken skin

In order to differentiate the contributions of scattering and birefringence in the phase retardation of polarized light propagated through the chicken skin, optical clearing^45^^,^^46^ was used to suppress scattering, whereas birefringence was induced by mechanical stretch. A separate measurement of the scattering properties of the chicken skin tissue with and without clearing was performed using the spectrophotometric system, as described in Sec. 2.2.1. Optical clearing was performed by applying 40% glycerol solution in water during 1 h. Alignment of collagen fibers in optically cleared chicken skin as a result of mechanical stretch was validated separately by the SHG imaging utilizing standard multiphoton microscope (A1R ${\mathrm{MP}}^{+}$, Nikon). The imaging was performed using CFI Plan Apochromat 10× G Glyc objective (corrected for water and glycerol) immersed in 40% glycerol-water solution without a cover glass.

Based on the findings acquired in the model experiments and SHG imaging of collagen fibers in chicken skin, three-stage experiments with samples of chicken skin were performed. The sample of chicken skin (size, $∼2.5\times 6.5\;{\mathrm{cm}}^{2}$) was excised from a chicken thigh and placed on a sample holder with the inner side of the skin up. To exclude the scattering on the roughness of skin and surface contaminations caused by flakes and/or fractions of residual feather follicles, the samples of chicken skin were measured from the inner side. The spatial scanning of the tissue sample was performed at the $2\times 2\;{\mathrm{mm}}^{2}$ surface area with a 200-μm step. The measurement at each scanning point was an average of 10 measurements. At the first stage, the sample was left intact for 30 min under normal conditions for reducing the level of humidity on the surface of the freshly excised sample. Further, the optical clearing agent (40% glycerol-water solution) was applied topically to the surface of the sample. After 60 min of optical clearing, the mechanical stretch (up to1.5 N) was gradually applied to the optically cleared sample along the plane of light incidence. The mechanical stretch was applied to the short end of the sample using gravitational force and a system of pulleys. No extra alignment of the sample was performed during the experiment in order to record the real-time polarization change during all three stages of the experiment without any external influence. The measurements were done every 5 min; the measured Stokes vectors were averaged throughout the scanning area and their changes in time were analyzed.

## 3 Results and Discussion

### 3.1 Model Experiments: Variation of Source–Detector Separation

**Fig. 2:**
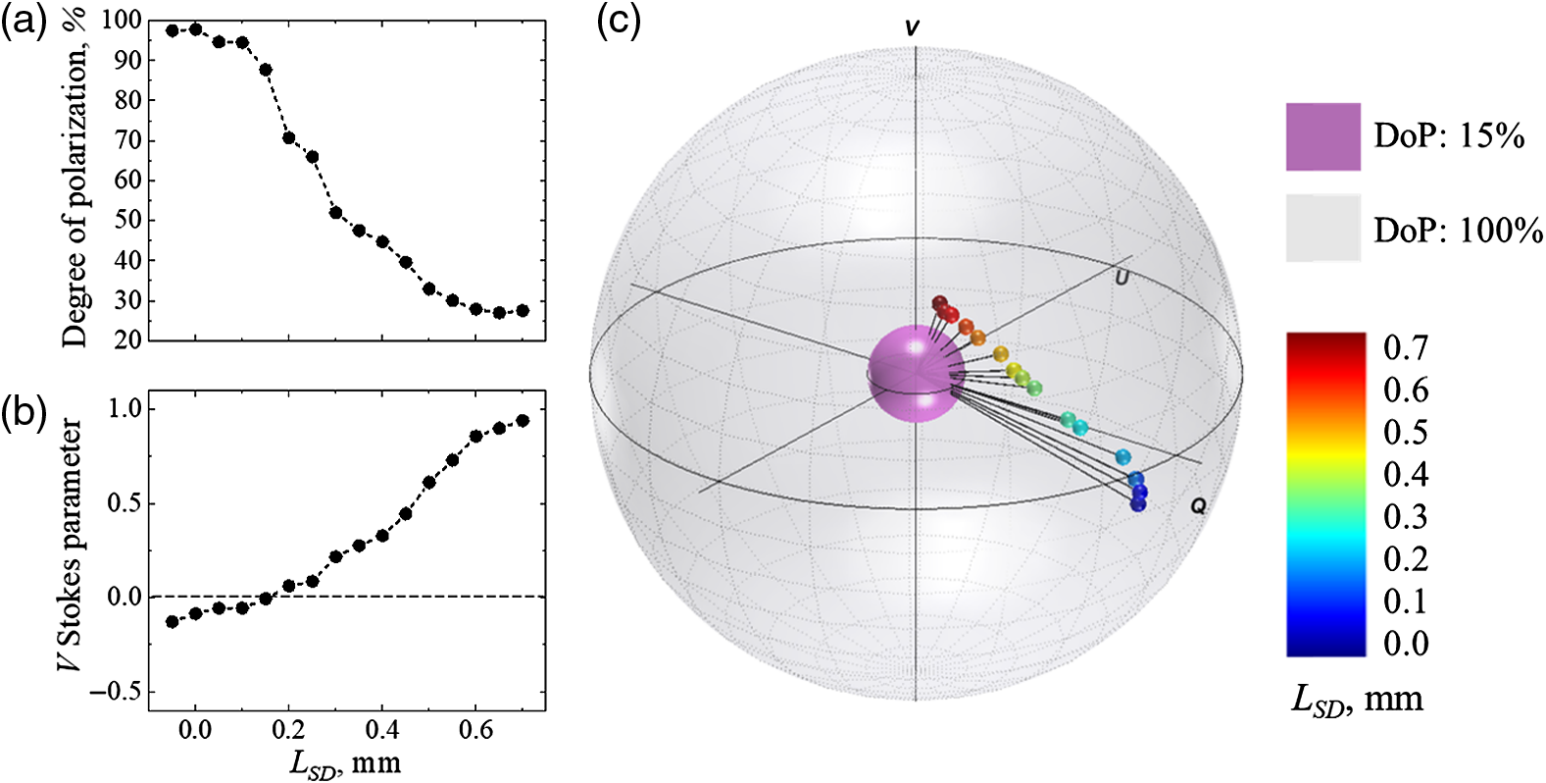
Impact of the variation of the source–detector separation on: (a) DoP, (b) V Stokes parameter, and (c) Stokes vector mapped on the Poincaré sphere with respect to the DoP. The radii of the outer (gray) and inner (purple) spheres correspond to 100% and 15% DoP, respectively. The color map from blue to red corresponds to the increase of the source–detector separation. Video 1 shows the Poincaré sphere rotating around V axis for better understanding of the positions of the Stokes vectors tips inside the Poincaré sphere (Video 1, mp4, 4 Mb [URL: https://doi.org/10.1117/1.JBO.25.5.057001.1]).

In the experiment with variation of the source–detector separation, $L_{\mathrm{SD}}$, the Stokes vectors of light scattered from a tissue phantom were measured at different $L_{\mathrm{SD}}$. Results of the experiment are presented in Fig. 2: (a) degree of polarization (DoP), (b) V Stokes parameter, (c) Stokes vectors mapped on the Poincaré sphere with respect to the DoP. In panel (c), the radii of the outer (gray) and inner (purple) spheres correspond to 100% and 15%, respectively. The incident light polarization, 100% right circular (Stokes vector [1;0;0;1]), is located at the north pole on the surface of the outer Poincaré sphere. The black lines connecting the origin with the tips of the Stokes vectors correspond to the DoP. Video 1 (mp4, 4 MB) shows the Poincaré sphere rotating around V axis for the better understanding of the positions of the Stokes vectors tips inside the Poincaré sphere.

The results in Fig. 2 show that the measured state of polarization of light scattered from the sample depended significantly on the separation between the source and detector. With small source–detector separation ($-0.05\;\mathrm{mm}\leq L_{\mathrm{SD}}\leq 0.15\;\mathrm{mm}$), the helicity of the detected light was left-handed [see Figs. 2(b) and 2(c): the Stokes vector is in the lower hemisphere as V Stokes parameter is negative], while the incident polarized light helicity was right-handed, which means that the majority of detected photons had flipped helicity after scattering from the sample. Due to the directional awareness of the circularly polarized light, the helicity flip is an indication of one or other odd number of backscattering events.^47^ Oppositely, for the larger source–detector separation ($0.2\;\mathrm{mm}\leq L_{\mathrm{SD}}\leq 0.7\;\mathrm{mm}$), helicity of light scattered from the sample was preserved, which indicates that the majority of photons underwent forward scattering.

The DoP of the detected light has changed significantly due to variation in $L_{\mathrm{SD}}$ [see Figs. 2(a) and 2(c)]. The highest values of the DoP (up to 97%) correspond to the smallest $L_{\mathrm{SD}}$, as in this configuration, the detected light underwent the least number of scattering events, which depolarize light. With the growing value of $L_{\mathrm{SD}}$, the DoP was decaying due to the growing contribution of multiply scattered photons. The results of these experiments demonstrate that this approach allows observing the effect of helicity flip described in the literature^24^^,^^36^^,^^47^^–^^50^ by variation of the scattering multiplicity of the incident polarized light.

### 3.2 Model Experiments: Phase Alterations Due to Scattering and Birefringence

**Fig. 3:**
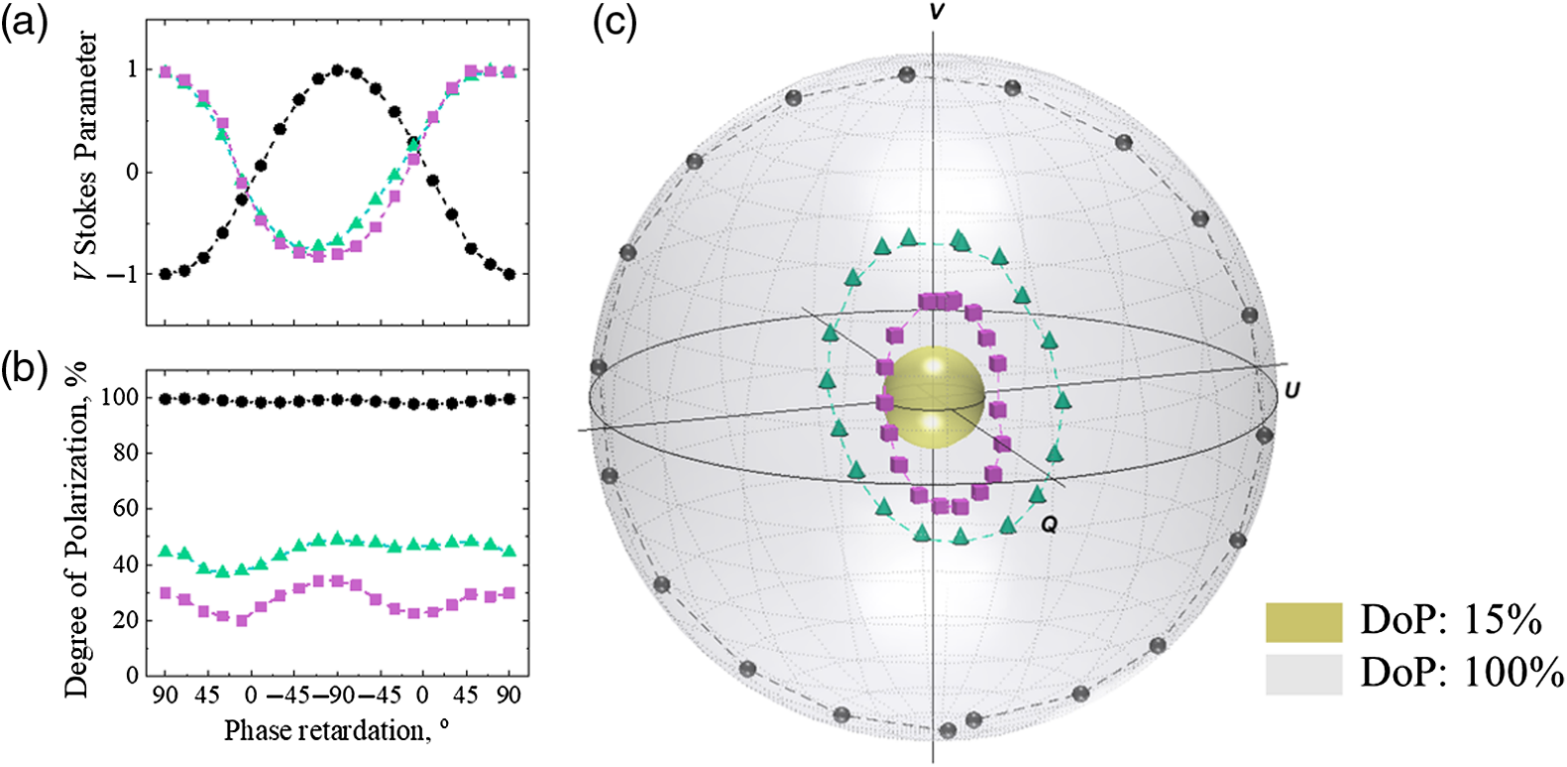
Results of the model experiments: (a) V Stokes parameter, (b) DoP of light reflected from the mirror (black circles, $\mu_{s}=0\;{\mathrm{mm}}^{-1}$) and scattered from two phantoms with different scattering coefficients (green triangles: $\mu_{s}=4\;{\mathrm{mm}}^{-1}$, purple squares: $\mu_{s}=8\;{\mathrm{mm}}^{-1}$), (c) Stokes vectors (black spheres, $\mu_{s}=0\;{\mathrm{mm}}^{-1}$, green cones: $\mu_{s}=4\;{\mathrm{mm}}^{-1}$, purple cubes: $\mu_{s}=8\;{\mathrm{mm}}^{-1}$) mapped on the Poincaré sphere with respect to the DoP. Inner (yellow) and outer (gray) spheres correspond to 15% and 100% DoP, respectively. Video 2 demonstrates the Poincaré sphere from panel (c) rotating around V Stokes axis (Video 2, mp4, 4 Mb URL: https://doi.org/10.1117/1.JBO.25.5.057001.2]).

In order to explore alterations of the phase of circularly polarized light due to changes in scattering and birefringence, experiments with a mirror (no scattering) and two scattering phantoms were performed. The state of polarization of light reflected from the mirror and backscattered from the phantoms with different scattering coefficients ($\mu_{s}=4$ and $8\;{\mathrm{mm}}^{-1}$) is shown in Fig. 3: (a) V Stokes parameter, (b) DoP, and (c) Stokes vectors mapped on the Poincaré sphere with respect to the DoP. The incident light polarization, right circular, corresponds to the north pole on the Poincaré sphere and 90 deg phase retardation between orthogonal polarization components. Video 2 (mp4, 4 MB) demonstrates the Poincaré sphere from panel (c) rotating around V Stokes axis for the better understanding of location of the Stokes vectors inside the Poincaré sphere.

As it is shown in Figs. 3(a) and 3(c), the phase alterations caused by birefringence led to the rapid change in the Stokes vector, whereas the DoP remained nearly the same for both phantoms [see Figs. 3(b) and 3(c)]. The change in the phase shift between two orthogonal electric field components of polarized light is observed as a translation of the Stokes vector on the surface of the Poincaré sphere with the corresponding fixed radius, whereas the phase change due to difference in scattering is observed as the difference of the radii of Stokes vector tracks within the Poincaré sphere. Based on these results, the experiments with chicken skin aiming at the observation of separate contributions of scattering and birefringence were performed. The relation of the model experiment with tissue phantom is extended to the experiment with skin tissue stretching in frame of the successive addition of phase alterations due to scattering to the phase shift coursed by birefringence within the resultant phase shift of the polarized light.

### 3.3 Contributions of Scattering and Birefringence in Phase Alterations Observed in Chicken Skin

In the experiments with chicken skin, scattering was reduced by optical clearing, whereas form birefringence was induced by applying mechanical stretch to the chicken skin sample. An independent measurement of the optical properties of chicken skin tissue with and without optical clearing using a spectrophotometric system^43^^,^^44^ has shown that after optical clearing, the scattering coefficient of chicken skin $\mu_{s}$ has decreased by 30% (from 13 to $9\;{\mathrm{mm}}^{-1}$), whereas the anisotropy factor g has increased by 18% (from 0.8 to 0.95).

**Fig. 4:**
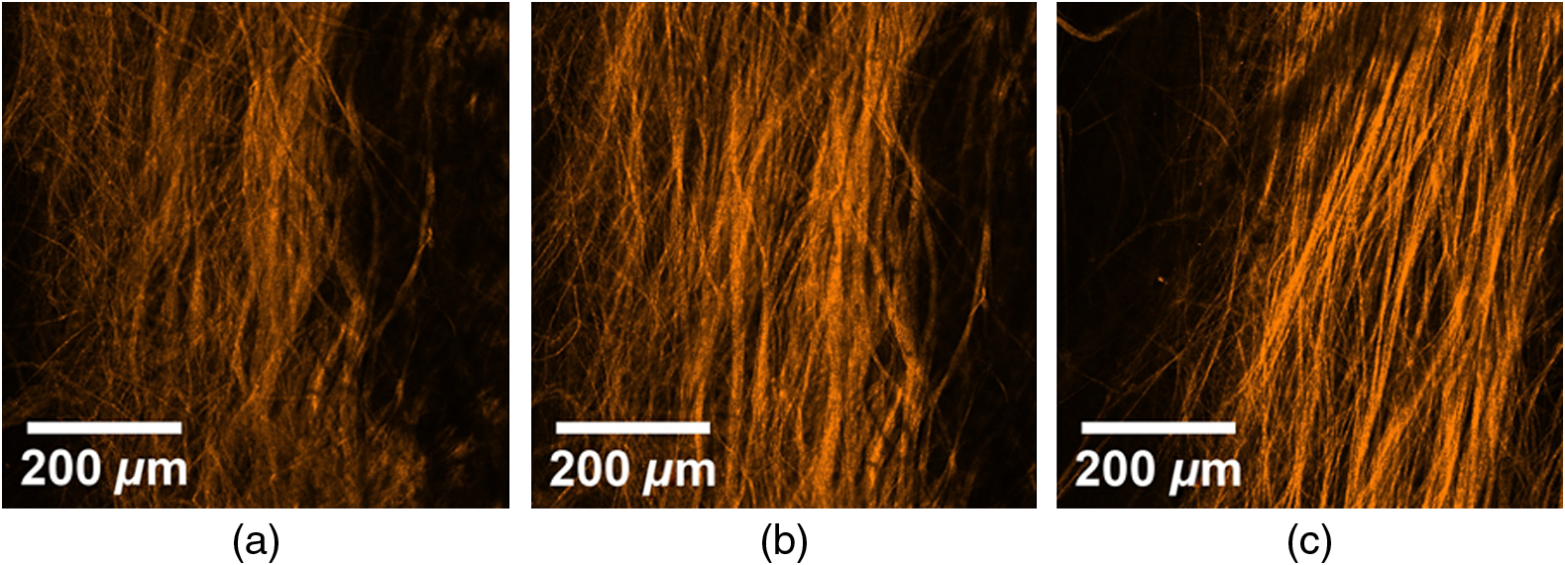
SHG microscopy of collagen fibers in the optically cleared sample of chicken skin with (a) no stretching, (b) stretching force of 0.74 N, and (c) stretching force of 1.35 N.

The alignment of collagen fibers in optically cleared chicken skin sample due to mechanical stretch was validated using the SHG imaging. Figure 4 illustrates SHG imaging of collagen fibers of roughly the same area of the sample influenced by different degrees of stretch: (a) no stretch, (b) stretching force of 0.74 N, and (c) stretching force of 1.35 N. As one can see in Fig. 4(a), the collagen bundle without any applied stretch was dispersed; however, after 0.74 N of stretch, it became more aligned and the SHG signal became brighter [see Fig. 4(b)]; after 1.35 N of stretch, this tendency became more prominent [see Fig. 4(c)]. The higher contrast of the fibers at higher degrees of stretch in SHG images correlates with the stronger SHG signal and additionally indicates the higher alignment of the fibers. The direction of the fiber alignment coincided with the direction of the applied stretching force.

**Fig. 5:**
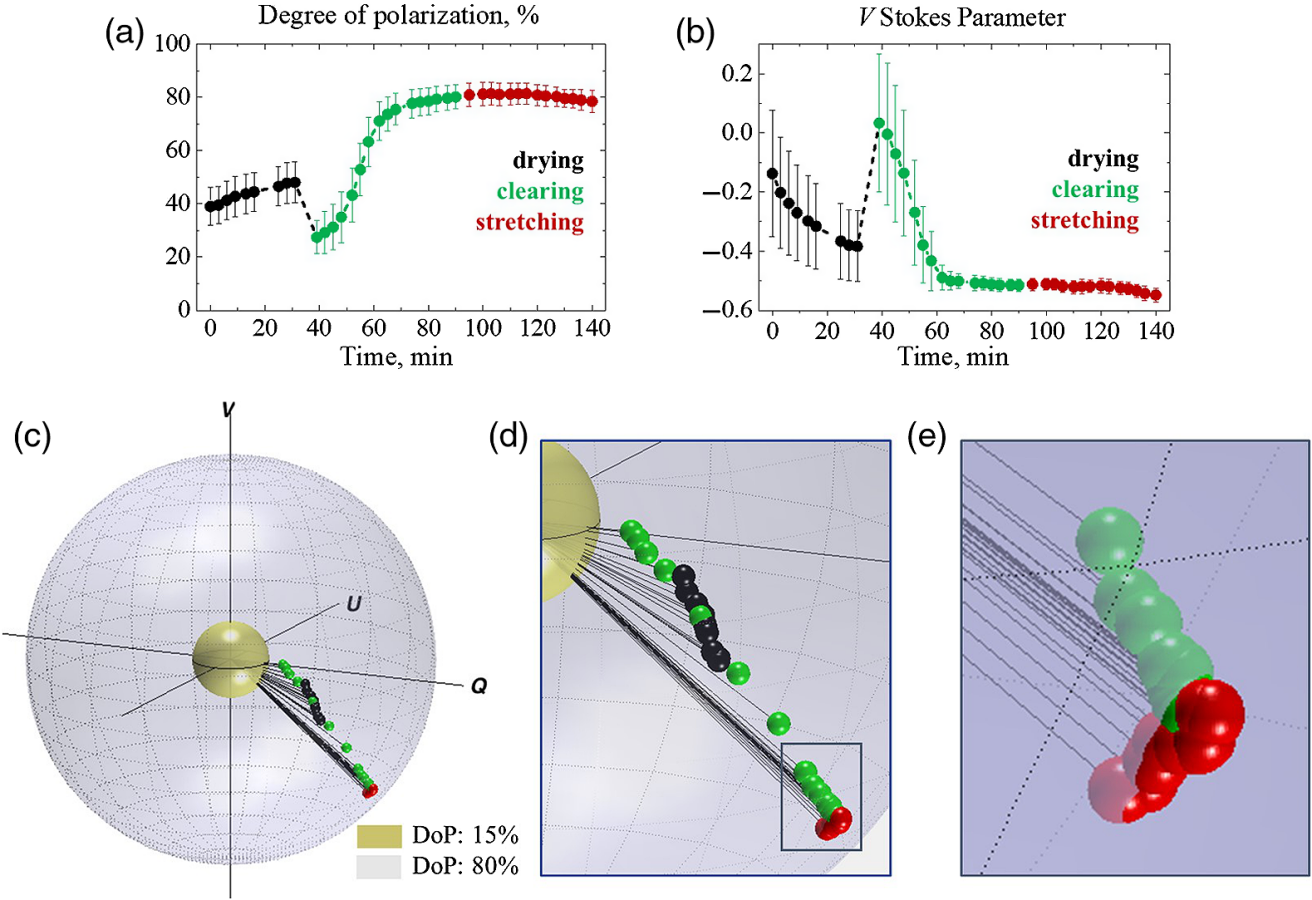
Alterations of the state of polarization of circularly polarized light scattered from the sample of chicken skin influenced by being kept under normal conditions (black), optical clearing (green), and mechanical stretch (red): (a) DoP, (b) V Stokes parameter, (c) trajectory of the Stokes vector plotted on the Poincaré sphere, (d) enlarged view of the Stokes vector track, and (e) close view of data points corresponding to stretch. Inner (yellow) and outer (gray) spheres correspond to 15% and 80% DoP, respectively.

Following the results of model experiments and spectrophotometric and SHG measurements of chicken skin, the three-stage experiments with samples of chicken skin were performed. The alterations of the state of polarization of light propagated within the sample of chicken skin being kept under normal conditions for 30 min (drying) and influenced by optical clearing during 1 h and mechanical stretch (up to 1.5 N) are presented in Fig. 5. Panels (a) and (b) show alterations of the DoP and V Stokes parameter in time; panel (c) illustrates trajectory of measured Stokes vector mapped on the Poincaré sphere: inner (yellow) and outer (blue) spheres correspond to 15% and 80% DoP, respectively; panel (d) shows an enlarged view of the Stokes vector track mapped on the Poincaré sphere; panel (e) shows closely the data points that correspond to the stretching. Each of the data points corresponds to the value of the Stokes vector component averaged over the scanning area ($2\times 2\;{\mathrm{mm}}^{2}$, 200-μm step, 10 measurements at each step) and the error bars represent the standard deviation. For the details of the Q and U Stokes vector components, refer to Fig. S1 in the Supplemental Material.

Following the results of experiments with variation of the source–detector separation (Sec. 3.1), the $L_{\mathrm{SD}}$ was set to 0.3 mm, as this was the largest value of separation that provided sufficient DoP (at least 40%). Sufficient DoP in the beginning of the experiment was necessary as the experimental protocol did not allow any extra alignment of the sample during the measurements in order to record the real-time polarization change without any external influence. Alterations of the Stokes vector while the DoP was lower than 20% were not considered reliable.

As one can see in Fig. 5(a), the DoP was ∼40% in the beginning of the experiment. The process of drying caused a growth of the DoP up to 50%, which was likely due to the reduction of scattering of the tissue sample in virtue of its shrinking.^51^ Once the optical clearing agent was applied topically to the skin tissue, the DoP dropped significantly due to matching of the refractive index on surface of the medium and activation of the impact of photons with longer pathlengths in the tissue to the measured signal.^52^ Further, during the optical clearing, the DoP grew exponentially up to 80% until it stopped changing by 80th minute of the experiment. Subsequent application of the mechanical stretch did not cause sufficient change in the DoP, which correlates with the results of the model experiments (Sec. 3.2), as mechanical stretch changed predominantly birefringence on the background of suppressed scattering.

The changes of V Stokes parameter are shown in Fig. 5(b). As one can see, at the beginning of the measurements, the state of polarization of multiply scattered light was close to linear, which means that the detected portions of the light with left- and right-handed helicities were almost equal. The process of drying led to the steady decay of V Stokes parameter, followed by a jump at the moment the optical clearing agent was applied. The diffusion of the optical clearing agent into the skin tissue caused reduction of scattering with the exponential decay of V Stokes parameter until it became asymptotic by 80th minute of the experiment. The mechanical stretch led to the alteration of birefringence in the sample, which was manifested in the renewal of the V Stokes parameter decay [Figs. 5(c)–5(e)]. This correlates with the Stokes vector tracks observed in the model experiments (Sec. 3.2) on a smaller scale, as the sample’s birefringence is minor.

The alteration of the V Stokes parameter in the third stage of the experiment was caused by the fact that the mechanical stretch aligned initially dispersed collagen fibers in a major direction, inducing form birefringence. The linearly oriented structure of collagen fibers in skin could be considered as a system of long dielectric cylinders characterized by the difference in the effective refractive index (Δn) for the light polarized along and perpendicular to the cylinders in the model.^4^ This indicates that the birefringence induced with the mechanical stretch influenced the state of polarization of the light scattered from the tissue sample. As the incident light polarization was circular, it contained the equal portions of the light polarized in parallel and perpendicular directions with respect to the optic axis of the collagen fibers structure. The retardance of one of these polarization components influenced the ellipticity of the resultant polarization, which changed the value of V Stokes vector component.

Though in 2D graphs in Figs. 5(a) and 5(b), changes due to mechanical stretch do not appear significant, the mapping of the Stokes vector on the Poincaré sphere allows identifying the nature of these changes [Figs. 5(c)–5(e)] and distinguishing them from changes of the Stokes vector due to variation of scattering. Thus, the Stokes vector alterations associated with drying and optical clearing of the biotissue are manifested as a shift down on the Poincaré sphere accompanied by the increase in the Stokes vector’s magnitude due to simultaneous changes in V Stokes parameter and DoP [Figs. 5(c) and 5(d)], while the Stokes vector alteration due to change in birefringence is observed as a shift on the surface of the Poincaré sphere with the vector’s magnitude preserved [see Fig. 5(e)]. As one can see, the red data points belong to the surface of the same sphere, while the direction of polarization state alterations due to optical clearing (down and toward the surface of the outer sphere) is sufficiently different from the one due to stretching [along the radius of the outer sphere, see Fig. 5(e)]. These results agree well with the results of the model experiments in Sec. 3.2.

The obtained results show that the relative phase δ between two orthogonal polarization components of the electric field of the incident circularly polarized light has changed approximately by 30% as a result of drying, in 4.5 times due to optical clearing and by 1.3% due to mechanical stretch. Changes of the phase retardation during drying and optical clearing are attributed to the variation of scattering. As the scattering was significantly reduced by the optical clearing, the application of mechanical stretch led to the phase retardation associated particularly with the birefringence induced in the sample. According to the obtained results, the birefringence (Δn=δλ/2πl, where λ is the wavelength and l is the photons pathlength within the tissue up to 1 mm^53^) for the chicken skin sample is estimated at $0.3\times 10^{-3}$. The result agrees well with the results of alternative studies.^4^ The overall change in the value of birefringence during mechanical stretch ($|\Delta n_{1}-\Delta n_{2}|=\Delta \delta \cdot \lambda /2\pi l$, where Δδ is the change in the relative phase) is estimated as $3.7\times 10^{-6}$. In fact, the impact of scattering on the DoP and phase alteration prevails significantly the phase shift due to birefringence. Therefore, it is almost impossible to observe the phase changes due to birefringence in skin at normal conditions. In our case, with a reduction of scattering utilizing optical clearing and with enhancement of birefringence by stretching, we were able to observe and assess them.

Thus, the changes of directions of the V Stokes parameter and DoP [see Figs. 5(a) and 5(b)] are associated, respectively, with the changes of anisotropy of scattering of circularly polarized light and the changes of scattering and total internal reflection on the medium boundary due to optical clearing. The impact of scattering on the circularly polarized light was extensively studied and evaluated earlier.^22^^–^^24^^,^^54^^,^^55^ While the V Stokes parameter and DoP on their own do not bring notable information in terms of distinguishing contributions of scattering and birefringence, the resultant Stokes vector trajectory on the Poincaré sphere [see Fig. 5(c)] allows one to reveal the role of both scattering and birefringence in the total phase retardation.

## 4 Summary and Conclusions

The study is focused on the assessment of the isolated contributions of scattering and birefringence in the overall phase retardation of the circularly polarized light propagated through the tissue-like scattering medium. With the help of the model experiments utilizing tissue phantoms, the influence of source–detector separation on the polarimetric response of the medium has been demonstrated. Moreover, the alteration of phase of circularly polarized light due to scattering and birefringence was illustrated using tissue phantoms and chicken skin tissue. In the experiments with chicken skin, it has been found that the phase retardation between two orthogonal electric field components of the circularly polarized light associated with scattering alterations has changed approximately by 30% during 30 min of drying and in 4.5 times during 1 h of optical clearing with the use of 40% solution of glycerol in water. Phase retardation associated with the alteration of birefringence has changed by 1.3% when mechanical stretch up to 1.5 N was applied. The decrease of tissue scattering due to optical clearing enhances the DoP up to 80% that makes birefringence distinguishable on the background of the remaining scattering. Thus, the birefringence, induced by mechanical stretch, is observed as the shift of the Stokes vector on the surface of the Poincaré sphere, whereas reduction of scattering is manifested in the growing magnitude of the Stokes vector, which was validated with model experiments. The overall change in the value of birefringence due to mechanical stretch is estimated as $3.7\times 10^{-6}$. The value of birefringence in chicken skin is estimated to be $0.3\times 10^{-3}$, which agrees well with the known literature data.^4^

Thus, the isolated contributions of scattering and birefringence in the phase retardation of circularly polarized light propagated in biological tissues have been demonstrated with the help of tissue-mimicking phantoms and chicken skin *in vitro* with application of the optical clearing and mechanical stretch. The alignment of collagen fibers in chicken skin due to stretch and, therefore, inducement of birefringence were validated by the SHG imaging. The Poincaré sphere is suggested to be used as a graphical tool for observing the trajectories of the Stokes vector for subsequent functional (qualitative) and quantitative characterization of biological tissues and turbid tissue-like scattering medium. The described approach can be beneficial for the more advanced characterization of various types of malformations within biological tissues, e.g., based on combination of Stokes-vector and Mueller-matrix polarimetry. This would allow functional quantitative assessment of phase-dependent Mueller-matrix elements and their interpretation in terms of phase retardation between the electric field components due to scattering and/or birefringence.
