# Supplementary material for: Role of scattering and birefringence in phase retardation revealed by locus of Stokes vector on Poincaré sphere
Source: J Biomed Opt. 2020 May 20;25(5):057001. doi: 10.1117/1.JBO.25.5.057001 (PMC7238295; doi:10.1117/1.JBO.25.5.057001)
Supplement: Supplementary file 1 [file JBO_025_057001_SD001.pdf]

Supplementary Material

Role of scattering and birefringence in phase retardation revealed by locus of Stokes vector on Poincaré sphere

Mariia Borovkova[a,*], Alexander Bykov[a], Alexey Popov[b], Igor Meglinski[a,c,d,e,f,]**

[a]Optoelectronics and Measurement Techniques Research Unit, University of Oulu, P.O. Box 4500, Oulu, Finland, 90014

[b]VTT Technical Research Centre of Finland, Kaitoväylä 1, Oulu, Finland, 90590

[c]School of Engineering and Applied Science, Aston University, Birmingham, UK, B4 7ET

[d]School of Life and Health Sciences, Aston University, Birmingham, UK, B4 7ET

[e]Interdisciplinary Laboratory of Biophotonics, National Research Tomsk State University, Tomsk, Russia, 634050

[f]Institute of Engineering Physics for Biomedicine (PhysBio), National Research Nuclear University "MEPhI", Moscow, Russia, 115409

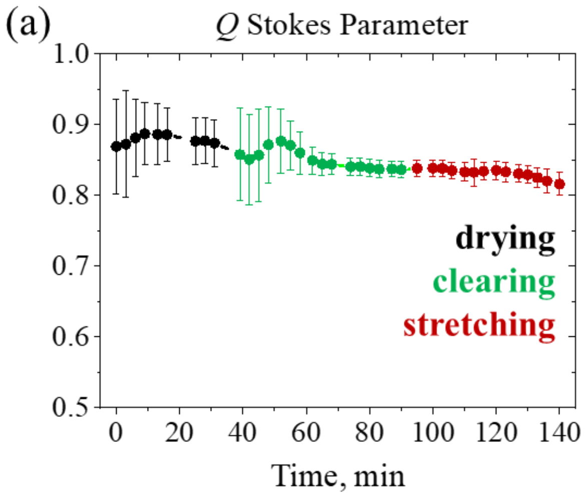

Fig. S1. Alterations of the *Q* and *U* Stokes vector components during drying, optical clearing and stretching of the sample of chicken skin.
